# Investigating the association between angina and osteoporosis: a 20-year longitudinal cohort study of ELSA

**DOI:** 10.1186/s12889-026-28218-y

**Published:** 2026-08-05

**Authors:** Ning Jiang, Lin Shen, Xiantie Zeng, Xinlong Ma

**Affiliations:** 1 Graduate School, Tianjin University of Traditional Chinese Medicine, Tianjin, 301617 China; 2 Foot and Ankle Surgery, Tianjin University Tianjin Hospital, Tianjin, 300211 China; 3 Tianjin University Tianjin Hospital, Tianjin, 300211 China

**Keywords:** Osteoporosis, Angina, Random forest model, Risk stratification analysis, ELSA

## Abstract

**Background:**

Osteoporosis (OP) is recognized as a major global health threat. Although an association between angina and osteoporosis was established, the underlying mechanisms connecting the two conditions were not clearly elucidated. Therefore, this study aims to systematically investigate the association between angina and OP of the English Longitudinal Study of Ageing (ELSA) database.

**Methods:**

This longitudinal cohort study utilized data from Wave 1 to Wave 11 of the ELSA database. Participants free of angina and OP at baseline were included. Time-dependent Cox proportional hazards regression models were employed to calculate hazard ratios (HRs) and 95% confidence intervals (CIs) for incident OP. Multiple imputation and rigorous sensitivity analyses (e.g., E-value, bootstrap) were performed to ensure robustness. Additionally, a random forest model was utilized to evaluate the relative importance of covariates, and the overall classification performance of the multivariable model was assessed using the area under the Receiver Operating Characteristic curve (AUC). OP was defined as a self-reported physician diagnosis with persistent confirmation, and angina was defined as a self-reported physician diagnosis.

**Results:**

A total of 6509 eligible participants were enrolled. During the follow-up, new-onset angina was significantly associated with an increased hazard of subsequent OP across all models. In the fully adjusted model, incident angina increased the risk of OP (HR = 1.621, 95% CI: 1.081–2.430, *P* = 0.02). Kaplan-Meier analysis demonstrated significantly lower OP-free survival probability in the new-onset angina group. Machine learning analysis confirmed time-dependent angina as a top predictor for incident OP. The AUC of the multivariable model was 0.793, indicating that the collective classification ability of all included variables was good.

**Conclusion:**

Among middle-aged and older adults initially free of these conditions, new-onset angina is an independent prognostic risk factor for subsequent osteoporosis. This longitudinal evidence clarifies the temporal relationship and highlights the need for OP surveillance in patients developing angina.

## Introduction

 Osteoporosis (OP) is a systemic skeletal disorder characterized by compromised bone strength and an elevated risk of fractures, ranking among the primary drivers of disability and mortality in the aging population [1, 2]. Despite the availability of antiresorptive and anabolic therapies, the clinical management of OP faces challenges, particularly regarding the intricate interplay of age-related comorbidities [3]. Concurrently, cardiovascular diseases exhibit a high prevalence in the elderly. While a growing body of research has explored the interplay between aggregate cardiovascular diseases and aberrant bone metabolism, attributing this to shared risk factors like aging, chronic inflammation, and declining estrogen levels [4, 5], there remains a critical gap in clinical epidemiological evidence. Most existing investigations have treated cardiovascular disease as a broad, homogenous category, largely overlooking the potentially differentiated impacts of specific cardiovascular phenotypes on the skeleton [6–8]. Therefore, it is imperative to specifically isolate and investigate angina—the most common clinical presentation of coronary atherosclerotic heart disease. Unlike the sustained tissue necrosis seen in myocardial infarction, angina is pathologically defined by acute, episodic, and reversible myocardial ischemia and hypoxia [9]. This unique pattern of intermittent ischemia-reperfusion may exert distinct cyclic effects on systemic bone metabolism [10]. Furthermore, patients with angina frequently require long-term antiplatelet therapy (e.g., clopidogrel), which preclinical studies have implicated in disrupting osteoclast and osteoblast function [11]. Despite this strong theoretical framework, robust population-based epidemiological data specifically targeting between the angina phenotype and OP remains sparse.To address the critical gap in temporal evidence, the present study utilized a longitudinal design from Wave 1 to Wave 11 of the ELSA. By strictly enrolling participants free of both angina and OP at baseline, this study aimed to investigate the temporal relationship and prospective risk of new-onset angina on subsequent incident OP. Through time-dependent Cox regression and rigorous sensitivity analyses, this work provides robust quantitative epidemiological evidence to unravel the temporal etiology linking angina and OP.

## Method

### Study design and data source

The ELSA is a nationally representative longitudinal cohort study supported by the UK Economic and Social Research Council. All non-sensitive data are available to qualified researchers via application through the UK Data Service platform. For the present longitudinal analysis, baseline data were extracted from Wave 1 (2002–2003) of the ELSA, and participants were prospectively followed up through Wave 11 (2023–2024).

### Study population and participant inclusion

**Fig. 1:**
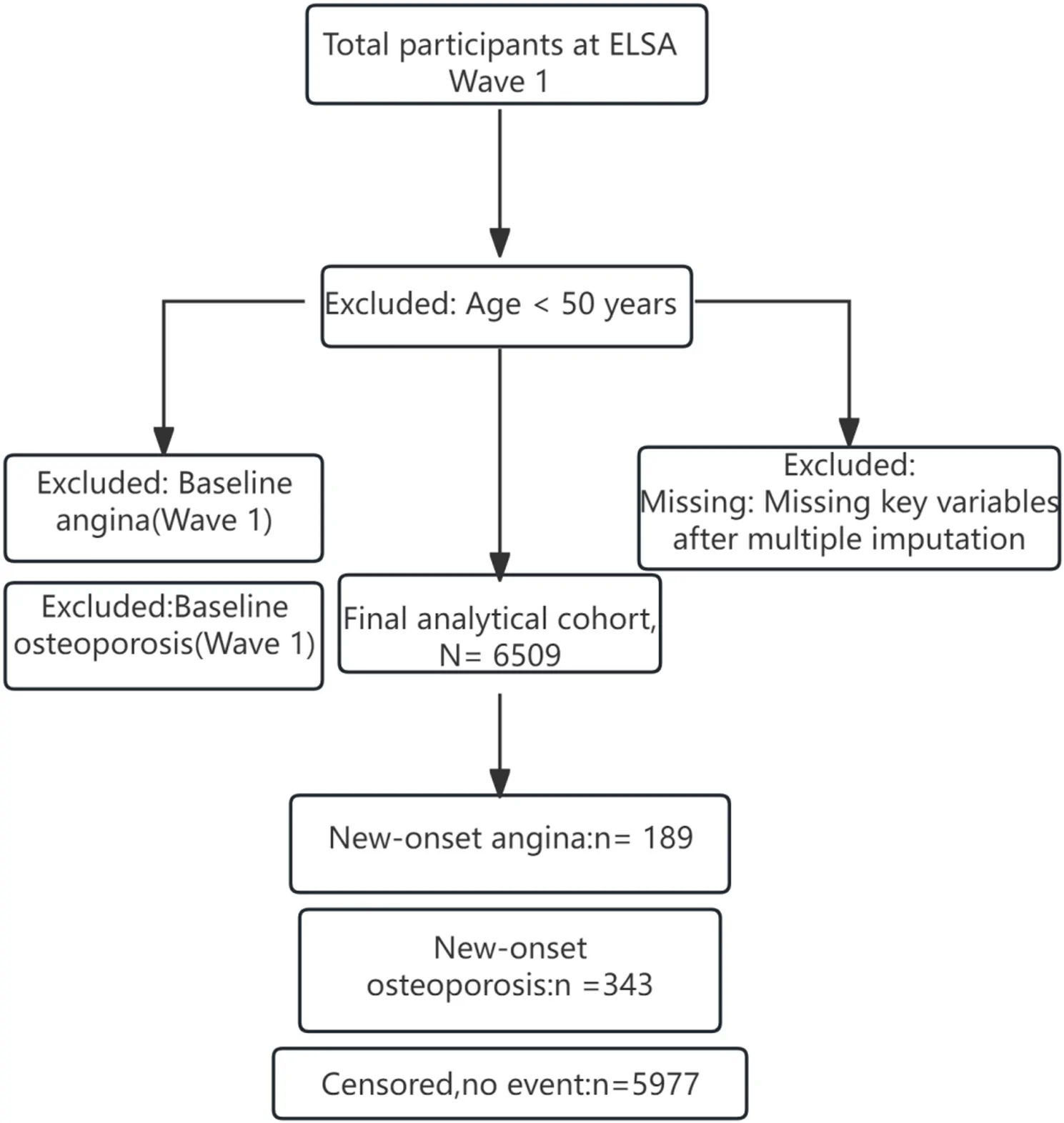
Flowchart of participant enrollment in the longitudinal cohort study

To establish a rigorous temporal sequence, we initially included participants aged ≥ 50 years at baseline. Crucially, participants who reported a history of angina or osteoporosis at baseline (Wave 1) were strictly excluded. Furthermore, individuals with missing key variables were excluded prior to imputation. The final analytical cohort comprised 6509 participants who were free of both angina and OP at baseline. The detailed participant enrollment and exclusion process is illustrated in Fig. 1.

### Data source

Data for the present study were extracted from the ELSA database, a large-scale, multidisciplinary prospective cohort study of adults aged ≥ 50 years residing in England. A nationally representative sample of middle-aged and elderly individuals was recruited using a stratified random sampling strategy. The cohort was established and maintained in strict compliance with international ethical guidelines, and written informed consent was obtained from all participants prior to study enrollment.This database captures comprehensive, repeated measurements collected at approximately 2-year intervals, encompassing data from face-to-face interviews, self-completed questionnaires, objective physical assessments, and biomarker analyses. The data scope spans multiple domains, including (but not limited to) health status, functional capacity, cognitive performance, mental health, social engagement, family composition, financial accumulation, and retirement planning.

### Definition of exposure and outcome

The primary exposure of interest was new-onset angina during the follow-up period. It was defined based on a self-reported physician diagnosis obtained during the follow-up waves (derived from the variable HEDIACAN). Specifically, participants were asked: “Has a doctor ever told you that you have angina?” Participants answering “Yes” were classified as having new-onset angina. Because angina could occur at any point during the 20-year follow-up, it was treated as a time-dependent covariate in the survival analyses to prevent immortal time bias.

The primary outcome was incident OP. Incident OP was purely defined by a self-reported physician diagnosis during the follow-up period (derived from the variables HEDIADOS and HEHAVEOS) (Table 1). Specifically, participants were asked: “Have you ever been diagnosed with osteoporosis by a doctor?” A “Yes” response established the diagnosis. Consequently, objective clinical thresholds (such as a Dual-energy X-ray Absorptiometry [DXA]-derived T-score ≤ -2.5) were not applicable and were not utilized in this study, as DXA-based bone mineral density measurements are not routinely collected across all ELSA waves.

**Table 1:** Variables and their identification information

Variable	Dataset and Definition Number
Age	INDAGER
Gender	INDSEX
Angina	HEDIACAN
Marital Status	DIMARR
Activity Level	HEACTA
Alcohol Consumption	SCAKO
Health Status	HEHELF
Smoking	HESMK
Osteoarthritis	HEARTOA, HEDIBAR

### Baseline characteristics of the participants

To characterize the baseline characteristics of the study population, data from Wave 1 (baseline) of the ELSA were utilized. Crucially, only participants completely free of both angina and OP at baseline were included in this summary. Baseline characteristics of the study population were summarized according to the presence or absence of OP. Categorical variables were presented as frequencies and percentages, and differences between groups were assessed using chi-square tests. It is important to note that these univariate comparisons were conducted for descriptive purposes only; the selection of covariates for multivariable models was strictly guided by clinical relevance and established literature rather than statistical significance (*P*-values). Differences in baseline characteristics between the two groups were assessed using the chi-square test, with statistical significance set at *P* < 0.05. A table summarizing baseline characteristics was generated using the “tableone” R package (version 0.13.2).

Covariate selection was strictly guided by clinical relevance and prior epidemiological literature. Baseline covariates included age, sex, marital status, physical activity level, alcohol consumption, smoking status, and self-rated health. Critically, socioeconomic status (SES)—a powerful confounder influencing healthcare access, lifestyle, and overall health—was incorporated into our analysis. Recognizing that key SES proxy indicators (e.g., economic wealth and educational attainment) exhibited high missing data rates (> 30%) across ELSA waves, we did not exclude these variables or delete cases, which could introduce severe selection bias. Instead, missing data were handled rigorously using multiple imputation by chained equations (MICE). Five imputed datasets (m = 5) were generated using the MICE package in R, incorporating all variables used in the analytical models to predict missing values. The pooled results across the five datasets were utilized for the final analyses, thereby allowing for the robust adjustment of SES without compromising statistical power.

### Statistical analysis

All statistical analyses were performed using R software (version 4.2.2). A two-sided *P*-value < 0.05 was considered statistically significant. The analytical workflow was structured as follows:

#### Descriptive analysis

Baseline characteristics of the strictly defined, disease-free cohort were summarized. Categorical variables were presented as frequencies and percentages, and continuous variables as means ± standard deviations. Differences in baseline characteristics stratified by the occurrence of incident OP were compared.

#### Kaplan-Meier survival analysis

Follow-up time was calculated from the baseline interview to the date of incident OP diagnosis, death, loss to follow-up, or the end of Wave 11. Kaplan-Meier survival curves were plotted to visually compare the OP-free survival probabilities between participants who developed new-onset angina and those who remained angina-free, with statistical differences assessed via the log-rank test. These analyses utilized the R packages dplyr (v1.1.4), survminer (v0.5.1), and survival (v3.8.3).

#### Time-dependent Cox proportional hazards regression

To quantify the prospective hazard of incident OP associated with new-onset angina, Time-dependent Cox regression models were utilized, reporting Hazard Ratios (HRs) and 95% Confidence Intervals (CIs). These analyses utilized the R packages survival, survminer, dplyr, broom (v1.0.10), and forestplot (v3.1.7). To transparently demonstrate the impact of confounders, three hierarchical models were constructed: Model 1 (Crude), unadjusted, including only the exposure (time-dependent angina) to present the baseline effect size. Model 2 (Partially adjusted model), adjusted for core demographic and structural covariates (age, sex, marital status, and imputed SES). And Model 3 (Fully adjusted model), further adjusted for lifestyle confounders (smoking and alcohol consumption). This stepwise adjustment strategy allows for a clear visualization of how different levels of confounders affect the magnitude of the association, yielding the most robust effect estimate. Prior to the final regression analysis, multicollinearity among all independent variables in Model 3 was rigorously assessed using the Variance Inflation Factor (VIF). The VIF values for all included covariates were consistently < 2 (well below the strict threshold of 5), confirming that no substantial multicollinearity existed to distort the regression results. Critically, based on causal inference frameworks, variables such as physical activity and self-rated health, which may lie on the causal pathway between incident angina and subsequent OP (acting as potential mediators rather than true confounders), were purposefully excluded from the final adjustment to avoid over-adjustment bias and appropriately estimate the total prognostic effect.

#### Sensitivity analyses

To rigorously validate the temporal robustness of the findings, four sensitivity analyses were conducted : (1) Bootstrap analysis with 1,000 replicates to verify the stability of the HR; (2) E-value calculation to assess how strong an unmeasured confounder would need to be to negate the observed association; (3) Leave-one-out cross-validation to ensure results were not driven by specific subsets; and (4) Comparison of models before and after multiple imputation to confirm consistency.

#### Machine learning and variable importance

To complement the Cox regression and evaluate the non-linear relative importance of covariates on the incident OP outcome, a random forest model was constructed using the randomForest package. Hyperparameter tuning was conducted via grid search to minimize Out-of-Bag (OOB) error, yielding optimal parameters of ntree = 98 and mtry = 7, with a fixed random seed (603) for reproducibility. The model relied on the algorithm’s inherent OOB internal cross-validation to prevent overfitting. Variable importance was ranked using the Mean Decrease Gini metric. Finally, the overall classification performance of this multivariable model was comprehensively evaluated using the AUC, alongside Accuracy, Sensitivity, and Specificity metrics. The machine learning analyses and visualizations were performed using the following R packages: randomForest (version 4.7.1.2), ggplot2 (version 3.5.2), dplyr, tidyr (version 1.3.1), tibble (version 3.3.0), and RColorBrewer (version 1.1.3).

## Results

### Baseline characteristics of the participants

Table 2 presents the baseline characteristics of the 6509 participants. Notably, all participants were 100% free of angina and OP at baseline. Table 3 summarizes the baseline characteristics and follow-up events stratified by the occurrence of incident OP.

**Table 2:** Baseline characteristics of the 6509 participants

Variable	Category	*N* (%) or Mean ± SD
Age (INDAGER)		64.2 ± 9.1
Gender (INDSEX)		
Male		2812 (43.2)
Female		3697 (56.8)
Marital Status (DIMARR)		
Married/cohabiting		4215 (64.8)
Single/divorced/widowed		2294 (35.2)
Activity Level (HEACTA)		
Low		1205 (18.5)
Medium		3124 (48.0)
High		2180 (33.5)
Alcohol Consumption (SCAKO)		
None		1892 (29.1)
Occasional		2603 (40.0)
Regular		2014 (30.9)
Smoking (HESMK)		
Never		3521 (54.1)
Former		2287 (35.1)
Current		701 (10.8)
Health Status (HEHELF)		
Good / Excellent		3892 (59.8)
Fair		2105 (32.3)
Poor		512 (7.9)
Angina (HEDIACAN)	Absent at baseline	6509 (100.0)
Osteoporosis	Absent at baseline	6509 (100.0)

**Table 3:** Baseline characteristics and follow-up events by new-onset osteoporosis status

Variable	Level	No OP group (*n* = 6166)	New-onset OP group (*n* = 343)	*P*-value
Sex	Female	3369 (54.6%)	300 (87.5%)	< 0.001
	Male	2797 (45.4%)	43 (12.5%)	
age_group	≤ 60	1625 (26.4%)	20 (5.8%)	< 0.001
	> 60	4541 (73.6%)	323 (94.2%)	
health	Excellent	660 (10.7%)	7 (2.0%)	< 0.001
	Fair	1147 (18.6%)	103 (30.0%)	
	Good	2117 (34.3%)	113 (32.9%)	
	Poor	445 (7.2%)	59 (17.2%)	
	Very good	1797 (29.1%)	61 (17.8%)	
marriage	Divorced	807 (13.1%)	47 (13.7%)	< 0.001
	Married, first and only marriage	3360 (54.5%)	162 (47.2%)	
	Remarried, second or later marriage	678 (11.0%)	31 (9.0%)	
	Single, that is never married	616 (10.0%)	32 (9.3%)	
	Widowed	629 (10.2%)	66 (19.2%)	
smoking	No	5702 (92.5%)	324 (94.5%)	0.208
	Yes	464 (7.5%)	19 (5.5%)	
drink	Almost every day	702 (11.4%)	33 (9.6%)	< 0.001
	Five or six days a week	371 (6.0%)	18 (5.2%)	
	Not at all in the last 12 months	876 (14.2%)	80 (23.3%)	
	Once every couple of months	481 (7.8%)	25 (7.3%)	
	Once or twice a month	744 (12.1%)	43 (12.5%)	
	Once or twice a week	1499 (24.3%)	68 (19.8%)	
	Once or twice a year	519 (8.4%)	42 (12.2%)	
	Three or four days a week	974 (15.8%)	34 (9.9%)	
activities	Hardly ever or never	3526 (57.2%)	260 (75.8%)	< 0.001
	More than once a week	1539 (25.0%)	51 (14.9%)	
	Once a week	589 (9.6%)	20 (5.8%)	
	One to three times a month	512 (8.3%)	12 (3.5%)	
new_angina_followup	No	5998 (97.3%)	322 (93.9%)	< 0.001
	Yes	168 (2.7%)	21 (6.1%)	

### Survival analysis (Kaplan-Meier)

**Fig. 2:**
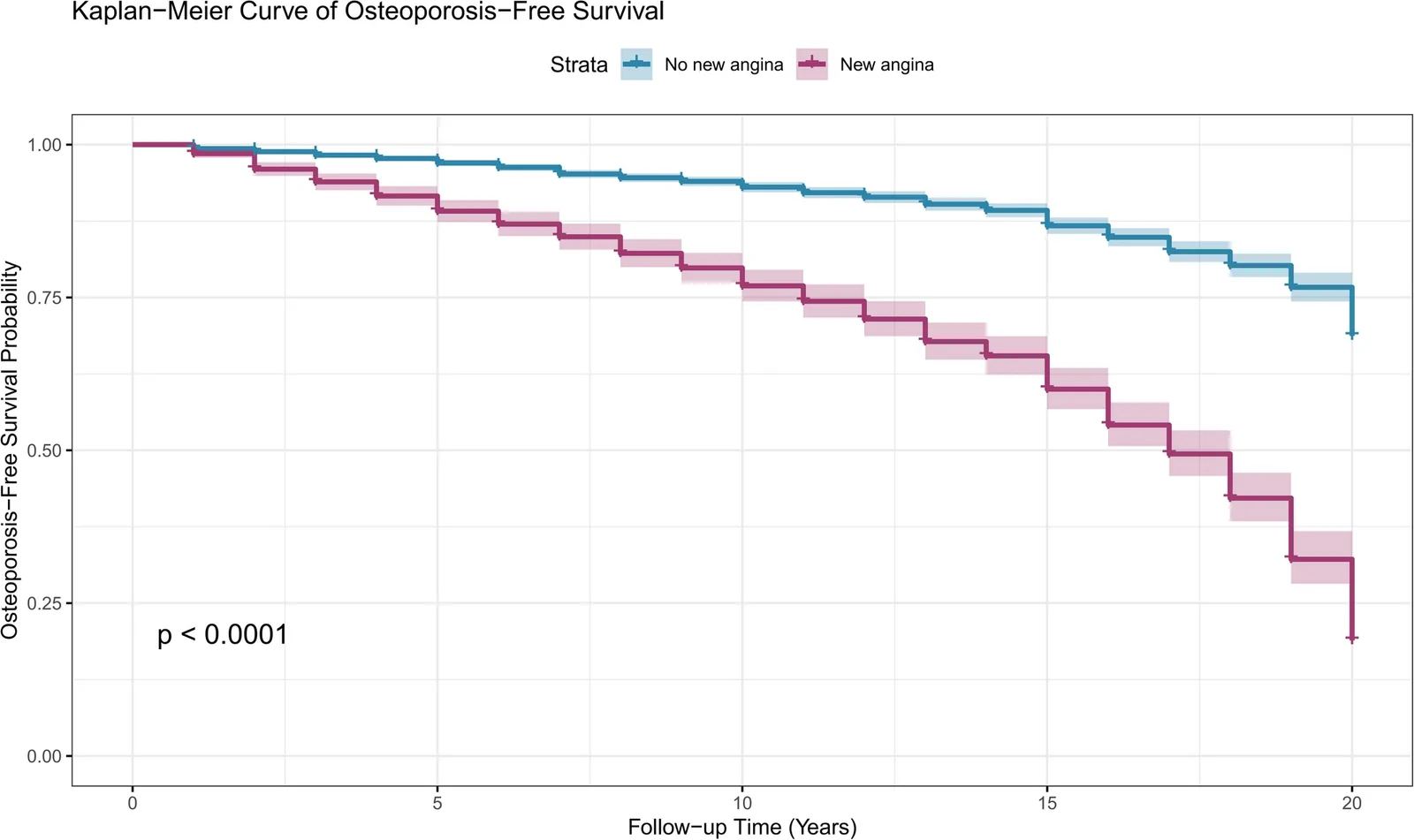
Kaplan-Meier curves of osteoporosis-free survival stratified by new-onset angina. The curves illustrate the probability of remaining free from incident osteoporosis over a 20-year follow-up period among participants initially free of both conditions at baseline. The blue line represents participants who did not develop angina (No new angina), while the purple/red line represents those who developed new-onset angina during follow-up (New angina). The shaded regions surrounding the curves denote the 95% confidence intervals, and the vertical tick marks indicate censored observations. The difference between the two strata was assessed using the log-rank test (*P* < 0.0001)

Kaplan-Meier survival analysis was conducted to visualize the temporal relationship. As illustrated in Fig. 2, the OP-free survival probability was significantly lower in participants who developed new-onset angina compared to those who remained angina-free throughout the follow-up period. The curves diverge progressively over time, strongly confirming the temporal precedence of angina prior to OP incidence (log-rank *P* < 0.0001).

### Time-dependent Cox proportional hazards regression

**Fig. 3:**
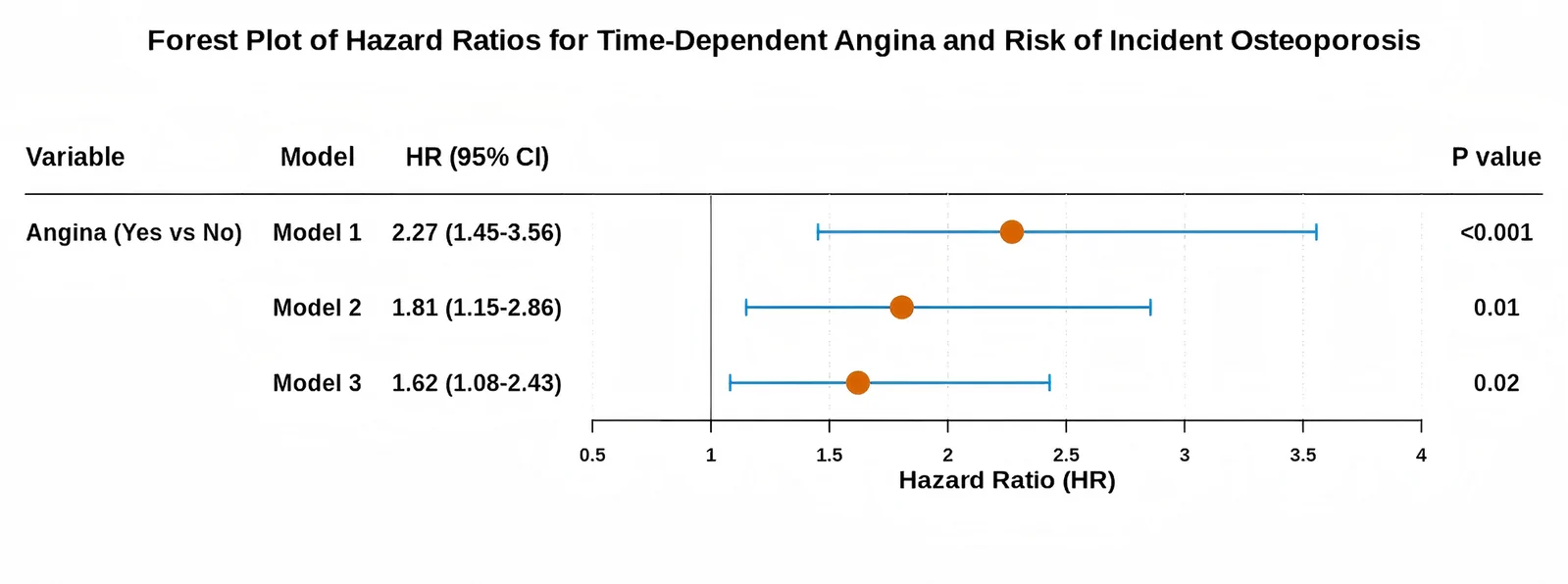
Forest plot of hazard ratios for incident osteoporosis associated with new-onset angina. The plot illustrates the risk of developing incident osteoporosis across three hierarchical time-dependent Cox regression models. Blue error bars represent 95% confidence intervals (CIs), and orange dots represent the Hazard Ratios (HRs). The vertical solid line at HR = 1 indicates no effect. Model 1 is unadjusted. Model 2 is adjusted for age, sex, socioeconomic status (SES), and marital status. Model 3 is further adjusted for smoking and alcohol consumption. The consistently significant HRs (all *P* < 0.05) across models demonstrate an independent longitudinal prognostic risk

Time-dependent Cox regression analyses unequivocally confirmed that new-onset angina significantly increases the risk of subsequent incident OP. In the crude model (Model 1), incident angina was strongly associated with incident OP (HR = 2.271, 95% CI: 1.452–3.557, *P* < 0.001). As expected in observational epidemiology, the sequential adjustment for true baseline confounders logically attenuated this hazard. After adjusting for core demographic and socioeconomic variables (including imputed SES) in Model 2, the hazard ratio was 1.806 (95% CI: 1.149–2.856, *P* = 0.01). In the fully adjusted Model 3, which further controlled for lifestyle confounders (smoking and alcohol consumption), the independent prospective risk remained robust and statistically significant (HR = 1.621, 95% CI: 1.081–2.430, *P* = 0.02). This stepwise attenuation confirms the confounding role of SES and lifestyle, while the persistently significant final HR definitively establishes new-onset angina as an independent prognostic risk factor for subsequent OP (Table  4; Fig. 3).

**Table 4:** Association between time-dependent angina and risk of incident osteoporosis using Cox regression models

		N	HR	95% CI	P
Angina	model 1	6509	2.271	(1.452–3.557)	< 0.0001
	model 2	6509	1.806	(1.149–2.856)	0.01
	model 3	6509	1.621	(1.081–2.430)	0.02

*Abbreviations*: *HR* Hazard Ratios, *CI* Confidence Interval

All three models utilized the same complete case cohort (*n* = 6509). Model 1: Crude model, unadjusted for any covariates. Model 2: Partially adjusted model, adjusting for age, sex, marital status, and socioeconomic status (SES). Model 3: Fully adjusted model, adjusting for age, sex, marital status, SES, smoking, and alcohol consumption

### Sensitivity analyses

Sensitivity analyses verified the stability of the primary findings (Table 5). The 1,000-replicate bootstrap analysis yielded consistent results (HR = 1.748, 95% CI: 1.031–2.840, *P* = 0.03). The calculated E-value lower bound was 2.32, indicating that a relatively strong unmeasured confounder would be required to negate the observed temporal association. Leave-one-out analysis and multiple imputation comparisons further confirmed the high robustness of the temporal risk.

**Table 5:** Sensitivity analyses for the association between angina and incident osteoporosis

Item	Results
Bootstrap analysis (1,000 replicates)	
HR (95% CI)	1.748 (1.031, 2.840)
*P*-value	0.03
E-value (lower bound)	2.32 (1.02)
Leave-one-out analysis	
HR range	1.702–1.791
Conclusion	Stable (no significant change)
Multiple imputation (m = 5)	
Before imputation HR (95% CI)	1.748 (1.031, 2.840)
After imputation HR (95% CI)	1.735 (1.026, 2.831)
Conclusion	Consistent before and after imputation

### Machine learning and model performance

**Fig. 4:**
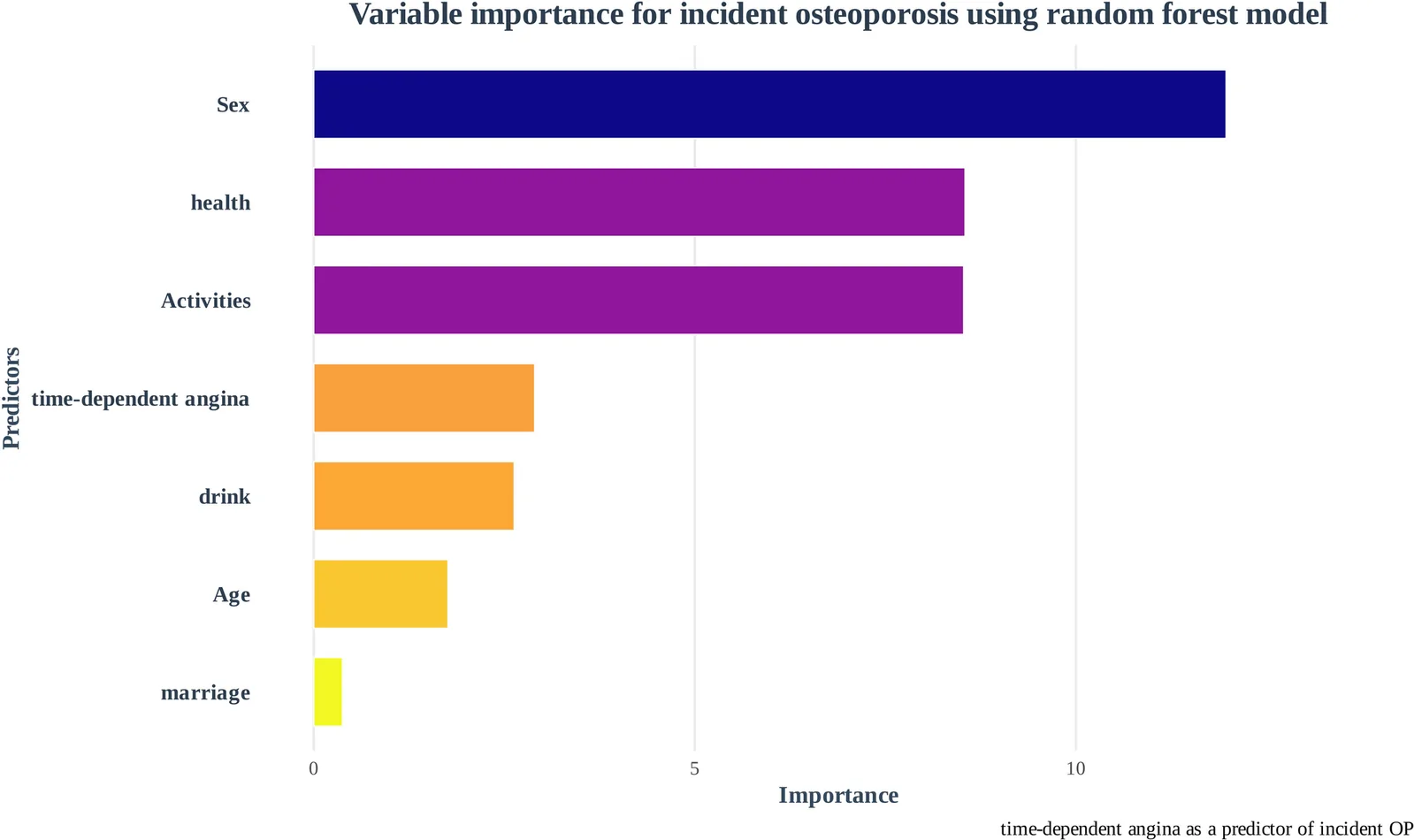
Machine learning. Each row represents one variable, and different lengths indicate different importance levels of the covariates - longer bars indicate higher importance

**Fig. 5:**
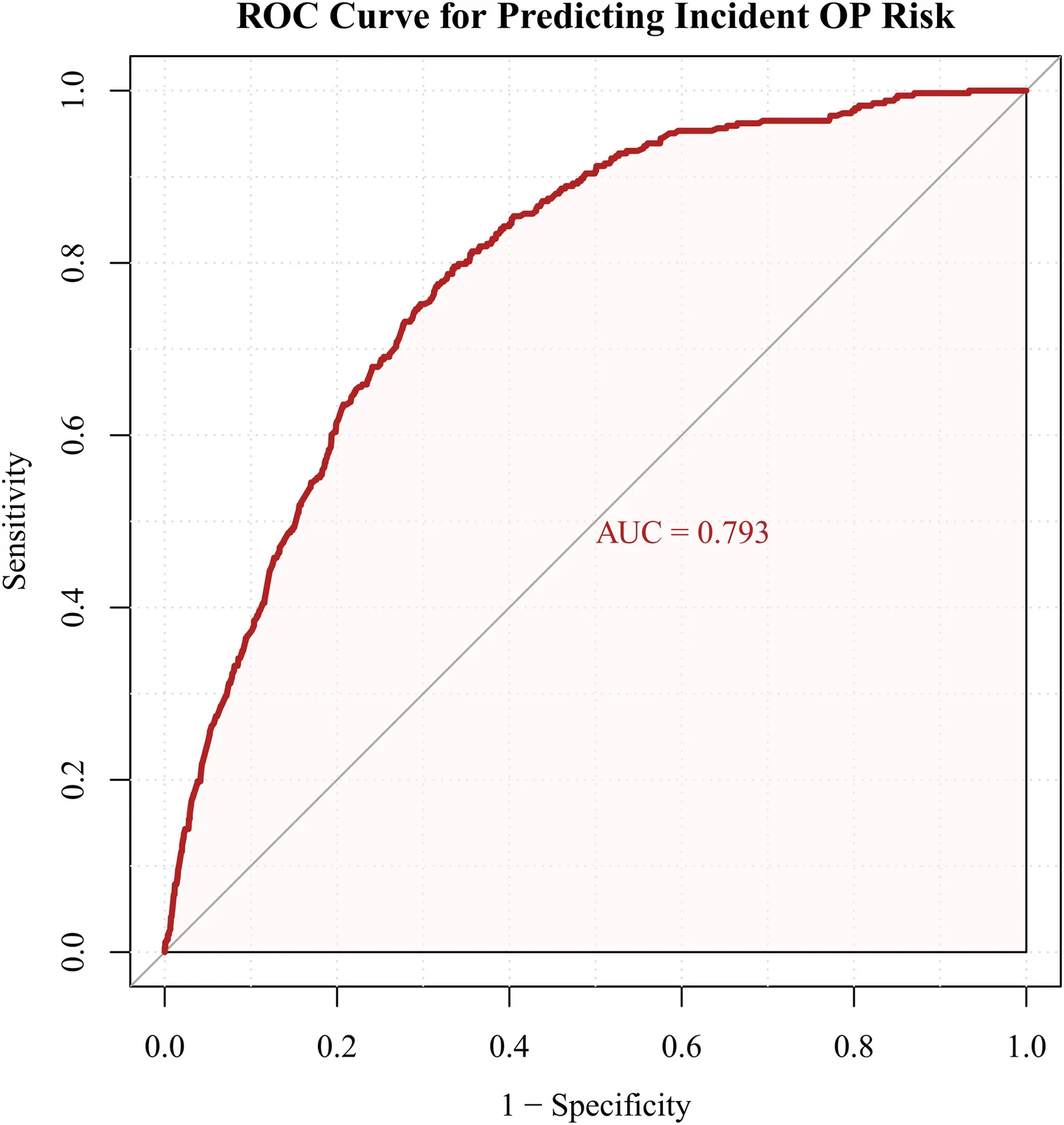
Receiver operating characteristic (ROC) curve of the multivariable random forest model. The x-axis represents 1-specificity (false positive rate), and the y-axis represents sensitivity (true positive rate). The red curve indicates the overall classification performance of the multivariable model (incorporating all selected covariates and angina) under different thresholds, achieving an AUC of 0.793. The diagonal line serves as a reference line for random classification

To complement the Cox regression, a random forest model ranked variable importance. As shown in Fig. 4, time-dependent angina emerged as a highly critical predictor for incident OP, ranking higher than traditional factors such as age and alcohol consumption. The comprehensive multivariable predictive model demonstrated a robust overall classification capability. Based on the internal OOB validation, the model achieved an AUC of 0.793 (Fig. 5), an overall Accuracy of 0.947, a Sensitivity (recall) of 0.175, and a highly robust Specificity of 0.990. The high specificity suggests the model is exceptionally effective at identifying individuals who will not develop incident OP. It is important to note that these metrics represent the collective performance of all selected covariates, rather than the isolated predictive value of angina.

## Discussion

OP is a degenerative skeletal disorder defined by reduced bone mineral density, impaired bone quality, and disrupted bone microarchitecture—pathological features that collectively culminate in heightened bone fragility and elevated fracture risk [12]. Despite advances in its clinical management, including targeted lifestyle modifications, pharmacotherapies, and surgical interventions, the epidemiological complexity of OP persists, and evidence derived from large-scale population-based datasets remains relatively limited [13]. The ELSA—a nationally representative, ethically compliant cohort with comprehensive multidimensional data—offers a robust platform for investigating OP-related risk factors, though its data analytics present non-trivial challenges. Leveraging 20-year longitudinal data from the ELSA, the present study established a definitive temporal sequence, demonstrating that new-onset angina is independently associated with an increased hazard of subsequent incident OP. A key finding was that angina remained independently associated with OP in middle-aged and elderly adults, even after rigorous adjustment for potential confounders (including age, gender, lifestyle factors, and self-rated health status). Notably, multiple analytical approaches—encompassing multi-model regression and machine learning—further validated the robustness of this association and clarified the relative importance of individual risk factors. These results provide novel epidemiological evidence to elucidate the comorbid relationship between cardiovascular conditions (specifically angina) and aberrant bone metabolism, addressing a critical gap in existing literature.

A major strength and original contribution of this study is its rigorous longitudinal design, which fundamentally resolves the ambiguity of reverse causality that plagues previous cross-sectional observations. By strictly limiting the analytical cohort to participants entirely free of both angina and OP at baseline (Wave 1), the design intrinsically guarantees the temporal sequence: the exposure (new-onset angina) definitively preceded the outcome (incident OP). Furthermore, the application of time-dependent Cox regression accurately captured the dynamic occurrence of angina during the follow-up, preventing immortal time bias. Kaplan-Meier survival curves visually reinforce this chronological trajectory, demonstrating a significantly accelerated decline in OP-free survival following the onset of angina (*P* < 0.0001). Moreover, to address the concern of shared etiology (e.g., aging, reduced physical activity), our hierarchical models progressively controlled for robust demographic, socioeconomic, and lifestyle confounders. The persistence of a strong effect size in the fully adjusted model strongly indicates that the temporal association between new-onset angina and subsequent OP is independent, rather than a mere artifact of shared vulnerabilities.

Having established a definitive longitudinal and temporal risk linking new-onset angina to subsequent OP, it is imperative to explore the potential underlying biological pathways. While our epidemiological data cannot directly confirm pathophysiological mechanisms, robust preclinical and clinical literature provides several plausible hypotheses for this temporal cascade. First, it is widely hypothesized that individuals with ischemic cardiovascular events experience chronic systemic inflammation and oxidative stress. Upregulated pro-inflammatory cytokines (e.g., IL-6, TNF-α) can directly disrupt bone homeostatic balance by promoting osteoclastic resorption [14–16]. Second, angina-induced transient and episodic myocardial ischemia might theoretically trigger a systemic redistribution of energy substrates toward the myocardium, potentially depriving bone tissue of the energy required for matrix synthesis over time [17–20]. Finally, the role of cardiovascular pharmacotherapies warrants consideration. Clopidogrel, frequently prescribed long-term for angina patients, has been implicated in suppressing osteoblast proliferation and inducing reductions in bone mineral density in both in vivo and observational studies [21–23]. Therefore, the strong prognostic risk observed in our longitudinal cohort is likely a composite manifestation of shared inflammatory pathways, microcirculatory dysfunction, and potential iatrogenic effects from antiplatelet therapies. Future translational studies directly measuring these biomarkers are required to validate these mechanistic hypotheses. Our longitudinal design, reinforced by time-dependent Cox regression and stringent sensitivity analyses (including E-values and multiple imputation for missing SES data), directly addresses this critical methodological gap, offering a highly original contribution to the epidemiological literature.

Our findings further identify gender as the strongest predictor of OP. A key underlying mechanism lies in age-related alterations in sex hormone levels: in women, estrogen exerts dual protective effects on bone homeostasis by inhibiting osteoclastic bone resorption and promoting osteoblastic bone formation, thereby sustaining BMD [24, 25]. However, postmenopausal women undergo a precipitous decline in endogenous estrogen, which disrupts this balance—accelerating bone resorption while impairing bone formation, and ultimately driving OP pathogenesis [5, 26]. In men, age-related testosterone decline also impacts bone health, but the magnitude of this effect is consistently smaller than that of postmenopausal estrogen loss in women [27]. Notably, estrogen additionally confers cardiovascular protection, with reduced endogenous estrogen levels in postmenopausal women linked to an elevated incidence of coronary heart disease [28, 29]. This dual role of estrogen creates a unique vulnerability: women—particularly postmenopausal women—face heightened co-risk of both OP and cardiovascular conditions, including angina [30, 31]. Collectively, these observations position sex—with females as the primary subgroup—as a critical shared risk factor bridging angina and OP. This insight not only reinforces the association identified in our study cohort but also underscores a clinical priority: elderly women with angina should be targeted as the key population for enhanced OP screening and personalized preventive interventions.

In conclusion, by utilizing a 20-year longitudinal cohort design, this study establishes a definitive temporal sequence demonstrating that new-onset angina significantly increases the hazard of incident osteoporosis. Addressing the fundamental logical limitations of previous cross-sectional observations, these findings robustly identify incident angina as an independent prognostic risk factor for subsequent bone loss. While causality cannot be absolutely confirmed without randomized controlled trials, this prospective epidemiological evidence strongly suggests that clinicians should be vigilant regarding bone health in aging patients newly diagnosed with angina.

Despite the robust longitudinal design, several limitations must be acknowledged. First, both OP and angina were assessed via self-reported new physician diagnoses. While practical in large-scale epidemiological cohorts like ELSA, the absence of objective clinical records or DXA-derived BMD data may introduce misclassification bias. Second, although we successfully utilized multiple imputation to adjust for socioeconomic proxy variables, residual confounding cannot be entirely ruled out. Specific critical variables, such as serum vitamin D levels, exact menopausal status, Body Mass Index (BMI), and detailed medication histories (e.g., specific antiplatelet usage durations), were unavailable in the dataset. However, our E-value sensitivity analysis (lower bound = 2.32) suggests that an unmeasured confounder would need a strong association with both angina and OP to completely explain away our findings. Future prospective studies integrating objective biomarkers are warranted to further refine these prognostic insights.To address these limitations and advance understanding, future studies should refine angina assessment by integrating objective clinical indicators (e.g., exercise stress tests, coronary imaging) with self-report to reduce misclassification bias, expand covariate adjustment to include socioeconomic factors (e.g., income, education) and biological markers (e.g., inflammatory cytokines, sex hormone levels) using advanced imputation techniques to handle missing data without excessive sample loss, and conduct longitudinal cohort studies or randomized controlled trials (e.g., evaluating angina treatment effects on OP outcomes) to establish causal links and provide high-level evidence for targeted OP prevention in patients with angina.

## Data Availability

The data that support the findings of this study are available from the ELSA. Restrictions apply to the availability of these data, which were used under license for this study. Data are available to qualified researchers upon reasonable request and with permission from the UK Data Service (https://www.ukdataservice.ac.uk/). The authors confirm that they did not have any special access privileges to the data that others would not have.
